# Simultaneous Routing with Washing Droplets Based on Shape-Dependent Velocity Model in MEDA Biochips

**DOI:** 10.3390/bios15080533

**Published:** 2025-08-14

**Authors:** Chiharu Shiro, Hiroki Nishikawa, Xiangbo Kong, Hiroyuki Tomiyama, Shigeru Yamashita

**Affiliations:** 1 Graduate School of Science and Engineering, Ritsumeikan University, Kusatsu 525-8577, Japan; chiharu.shiro@tomiyama-lab.org; 2 WITZ Corporation, Nagoya 460-0004, Japan; 3 Graduate School of Information Science and Technology, The University of Osaka, Suita 565-0871, Japan; nishikawa.hiroki@ist.osaka-u.ac.jp; 4 Department of Intelligent Robotics, Faculty of Information Engineering, Toyama Prefectural University, Imizu 939-0398, Japan; kong@pu-toyama.ac.jp; 5 College of Information Science and Engineering, Ritsumeikan University, Suita 567-8570, Japan; ger@cs.ritsumei.ac.jp

**Keywords:** digital microfluidics, biochips, MEDA, droplet routing, mathematical programming problem

## Abstract

Micro Electrode Dot Array (MEDA) biochips have recently attracted considerable attention in the biochemical and medical industries. MEDA biochips manipulate micro droplets for biochemical experiments such as DNA analysis. Droplets on MEDA biochips are moved using the Electrowetting on Dielectric (EWOD) effect, but a portion of a droplet may remain on a cell after passing through, contaminating the cell. Other droplets cannot pass through a contaminated cell. In previous studies, contaminated cells were considered unavailable for droplet routing. As the number of contaminated cells increases, droplets may be prevented from moving to the desired position. Therefore, we propose a method for simultaneous routing of target functional and washing droplets based on a shape-dependent velocity model. In a simulation, the proposed method reduced the routing time by about 10% compared with an existing method.

## 1. Introduction

Digital Microfluidic Biochips (DMFBs) are Lab-on-a-Chip (LoC) devices attracting attention in the fields of biochemistry and medicine [1,2,3]. However, DMFBs face challenges in terms of functionality and reliability for practical use, as they are unable to control the volume and shape of droplets during manipulation or detect droplets in real time [4,5]. The Micro Electrode Dot Array (MEDA) biochip was designed to address these challenges.

In DMFBs, a single cell consists of one electrode. In contrast, in MEDA biochips, a single cell is composed of a group of microelectrodes called Microelectrode Cells (MCs). Using MCs to control droplets enables fine adjustments of their size and shape—something that was not achievable with DMFBs [6,7]. Furthermore, real-time sensing has been made possible by integrating droplet-sensing circuits into MCs [8]. In DMFBs, the complexity of the mixing process results in longer processing times. Therefore, the time required for droplet routing before mixing is initiated is negligible compared to the overall processing time. However, MEDA biochips enable mixing at varying ratios, significantly improving the mixing and reaction processes. As a result, the time required for droplet routing can no longer be considered negligible [9].

When a droplet moves across the biochip, a portion of the droplet may remain in the cell it has passed through, potentially contaminating that cell. If a droplet passes through a cell that contains residual fluid from a previous droplet with different properties, the remaining droplet may affect the properties of the passing droplet. One method of eliminating the effects of residual droplets allows only a single type of droplet to pass through each cell, preventing interference, even if residual droplets remain [10]. Another method avoids interference by washing the cells as needed to remove any residual droplets [11,12,13]. These methods [11,12,13] have been implemented in DMFBs, but they cannot be applied to MEDA biochips, where a droplet’s moving velocity depends on its volume and shape [14]. Therefore, we propose a method that simultaneously routes functional droplets and washing droplets by utilizing the droplet moving velocity, which varies depending on droplet shape and volume.

The contributions of this study are as outlined as follows:

1. We developed a simultaneous routing method for multiple droplets, including washing droplets, on a MEDA biochip.
2. We realized a routing method that assumes that washing will make pathways previously blocked due to contamination available for droplet routing.
3. We solved a routing time minimization problem by considering the difference in droplet moving velocity, including washing with different volume ratios of functional and washing droplets.

The rest of this paper is organized as follows. In Section 2, we formulate the problem of simultaneous routing of functional and washing droplets using the droplet moving velocity, which depends on droplet shape and volume. Section 3 describes the experiments and presents a comparison of the results. Section 4 contains the conclusions of this study.

## 2. Related Works

Since the 2000s, extensive work has been conducted on efficient droplet routing in DMFBs [5,15]. However, DMFBs can only perform droplet mixing at a 1:1 volume ratio. In addition, droplet movement is restricted to the x and y directions [16]. Most work on DMFBs has been conducted under these constraints, and the impact of droplet routing time is considered small when dealing with complex mixing ratios.

**Figure 1:**
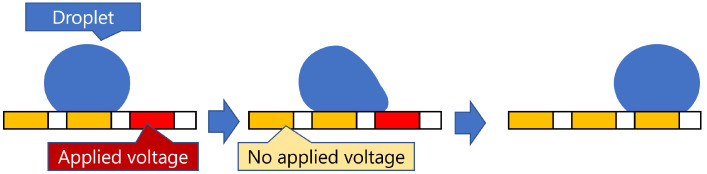
An example of droplet movement using the Electrowetting on Dielectric (EWOD) effect. Applying voltage to a dielectric-coated electrode reduces the contact angle of the droplet, increasing wettability. This creates a surface tension gradient that moves the droplet toward the activated electrode.

MEDA biochips have achieved a variety of droplet manipulations by dividing electrodes into microelectrodes (MCs) [17,18,19,20]. Droplets are moved using the electrowetting on dielectric (EWOD) effect, which is generated by actuating the microelectrodes (MCs). Figure 1 shows an example of moving a droplet using the electrowetting on dielectric (EWOD) effect. Each MC is equipped with a device that can detect and control droplets, making it possible to perform real-time droplet detection within 10 milliseconds, which was not achievable with DMFBs [8]. Furthermore, by grouping MCs, it is possible to manipulate droplets of various volumes, enabling mixing ratios other than 1:1 [6,7]. Dilution and mixing manipulations that require multiple steps in DMFBs can be performed in a single step by leveraging the features of MEDA biochips, significantly reducing the time required for each operation [9]. As a result, the impact of droplet routing time has become more significant than in DMFBs. Compared to DMFBs, MEDA biochips enable more diverse manipulations and offer greater flexibility; therefore, methods used in DMFBs are not necessarily optimal for MEDA biochips.

Droplets of a fixed volume on a MEDA biochip can be manipulated into various shapes by controlling the MCs. The force exerted on a droplet by a group of MCs acts on the portion of the droplet adjacent to the cell, so the droplet’s moving velocity depends on its volume and shape [14]. However, many previous works on MEDA biochips did not consider the shape-dependent droplet velocity, making their routing methods inefficient from the perspective of droplet velocity. Furthermore, when morphing the droplet into a different shape, the associated overhead must be taken into account [21,22].

When a certain number of cells become unavailable or when the number of droplets relative to the chip size makes it impossible to avoid interference with residual droplets, it becomes necessary to wash the biochip. In the former situation, the issue can be resolved by washing the chip before routing [23]. However, in the latter case, it is necessary to route both functional droplets, which are intended for operations such as mixing, and washing droplets at the same time. Some studies have addressed the simultaneous routing of functional droplets and washing droplets [11,12,13]. These works mainly focus on methods that wash droplets at crossing points that occur when routing multiple functional droplets. However, since these three works targeted DMFBs, they are not optimal for MEDA biochips due to the difference in droplet size between washing droplets and functional droplets. Therefore, in this study, we propose simultaneous routing of washing droplets and functional droplets on a MEDA biochip based on a shape-dependent velocity model as an integer programming problem.

## 3. Simultaneous Routing with Washing Droplets

### 3.1. Problem Description

This section formulates the problem of simultaneous routing of washing droplets and functional droplets on a MEDA biochip. In this context, the coordinates of each cell are defined as (x,y), with the bottom-left corner as the origin (1,1), forming a MEDA biochip with dimensions of *W × H*. The cells on a MEDA biochip can manipulate the volume of droplets. Droplets can morph into different shapes at any time, and their velocity depends on both their volume and shape [9]. In this case, the droplet’s velocity depends on the number of active cells adjacent to the droplet [9].

**Figure 2:**
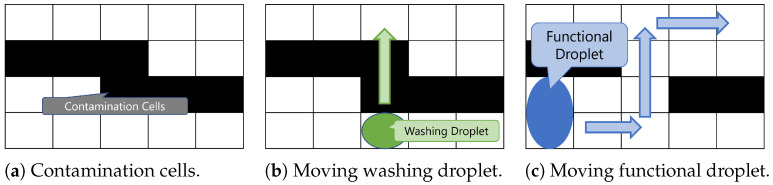
Examples of washing and functional droplet movement and contamination cells.

A MEDA biochip often contains unavailable cells due to MC degradation and contamination. The locations of unavailable cells are analyzed and known in advance. We assume a scenario in which some cells are contaminated and cannot be used, as shown in Figure 2a. The washing droplet (Figure 2b) and the functional droplet (Figure 2c) both move across the chip. The functional droplet moves from the bottom left to the top right of the biochip, while the washing droplet moves from the bottom edge toward the top edge. The washing droplet is assigned any one cell at the edge of the chip as its start cell and goal cell.

One of the functions of a MEDA biochip is the ability to morph droplets. Morphing a droplet requires one unit of operation time, but depending on the direction of movement, it can reduce the number of cells that the droplet must pass through, offering a potential advantage.

**Figure 3:**
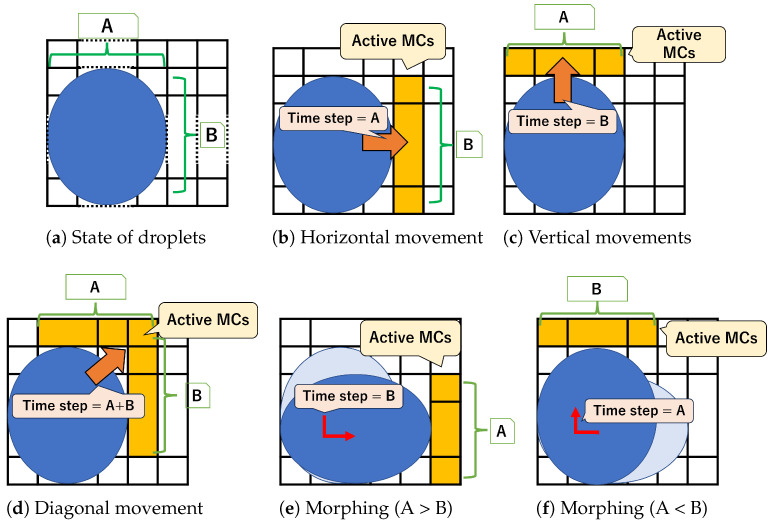
Movement and active MCs on MEDA biochips. (**a**) shows droplet size is A×B. (**b**) shows example of active MCs and moving *A* time step, when moving horizontal. (**c**) shows example of active MCs and moving *B* time step, when moving vertical. (**d**) shows example of active MCs and moving A+B time step, when moving diagonal. (**e**) shows example of active MCs and moving *B* time step, when morphing from A×B to B×A.(**f**) shows example of active MCs and moving *A* time step, when morphing from B×A to A×B.

Next, we describe the time required for each motion. Figure 3 shows droplet movement and active MCs on a MEDA biochip. The velocity of the droplet is proportional to the number of cells adjacent to it in the direction of movement and inversely proportional to its volume [9]. For a droplet of size A×B (Figure 3a), the time required for movement is defined as one time step when A×B adjacent cells are activated. Figure 3b shows an example of a droplet of size A×B moving horizontally. *B* adjacent cells are activated, and the movement takes *A* time steps. Similarly, Figure 3c shows that if the droplet moves vertically, *B* time steps are required. Figure 3e,f show a morphing droplet. When morphing from A×B to B×A, if *B* adjacent cells are activated, it takes *A* time steps (Figure 3e). Similarly, when morphing from B×A to A×B, if *A* adjacent cells are activated, it takes *B* time steps (Figure 3f). Diagonal movement is specifically defined as a combination of horizontal and vertical movements. Therefore, while the number of adjacent active MCs is A+B−1, the time required for movement is A+B time steps (Figure 3d).

In this study, we solve the routing problem to minimize the routing time of both functional and washing droplets.

### 3.2. Example

**Figure 4:**
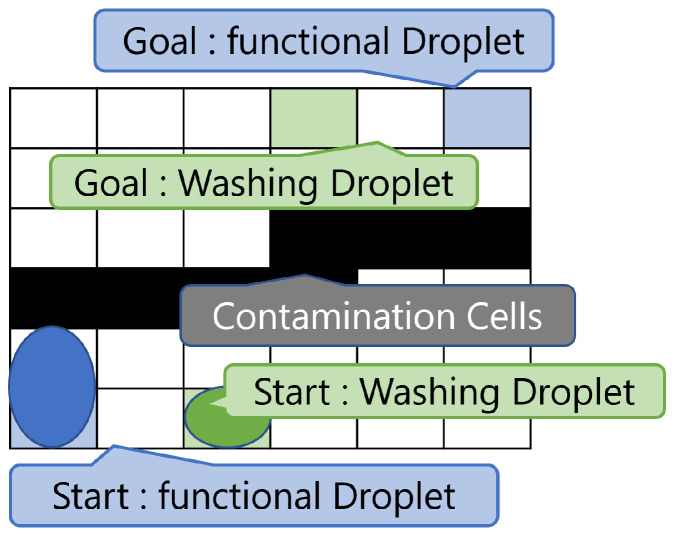
Example of initial biochip state. The biochip size is 6×6. The functional droplet size is 2, and it starts from (1,1) and moves to (6,6). Washing droplet size is 1, and it starts from (3,1) and moves to (4,6). Contamination cells are given randomly.

We consider a problem in which pre-existing contaminated cells that can be washed are present, and a size-1 washing droplet and a size-2 functional droplet are routed simultaneously. The start and goal cells of the washing droplet are given as inputs. Figure 4 shows a 6×6 biochip, indicating the contaminant cells, the start and goal cells of each droplet, and the initial shapes of the droplets at the start. Both the washing and functional droplets move from their respective start cells to their goal cells. When the washing droplet reaches its goal, it is discharged into a reservoir and no longer exists on the biochip. Because there is a possibility of interference between the washing and functional droplets, a certain distance must be maintained between them during movement. The contaminated cells, shown as black cells in Figure 4, are washed when the washing droplet passes through them, making those cells available for use from the next operation timing. The objective of this problem is to minimize the time required for both the functional droplet and the washing droplet to reach their respective goal cells. In this section, the *t* variable is used to denote time steps. For example, t=1 corresponds to a time step of 1.

**Figure 5:**
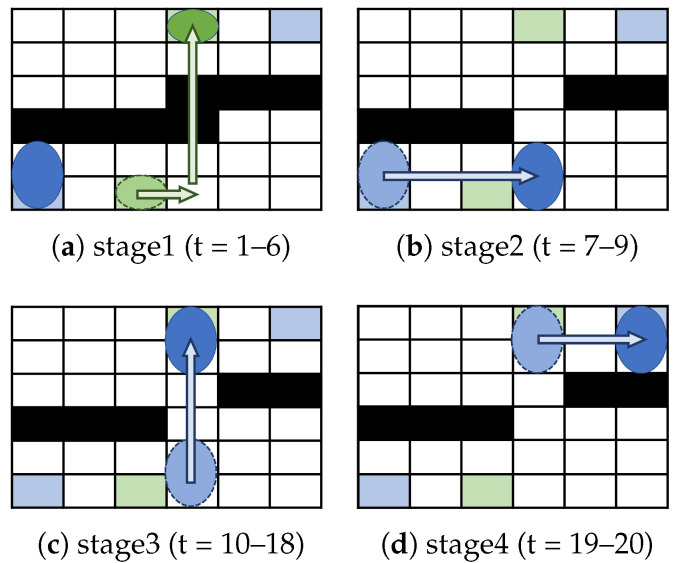
Example of Existing Method 1 [23]: (**a**) droplet routing step from time step 1 to time step 6; (**b**) droplet routing step from time step 7 to time step 9; (**c**) droplet routing step from time step 10 to time step 18; (**d**) droplet routing step from time step 19 to time step 20.

First, we calculate the routing time based on Existing Method 1 [23]. Existing Method 1 optimizes the paths of the washing and functional droplets separately and does not assume simultaneous routing with the functional droplet. In other words, the existing method determines the path of the functional droplet only after the washing droplet has finished washing the cell. As shown in Figure 5a, the washing droplet moves, requiring six time steps for its movement. Then, as shown in Figure 5b–d, the functional droplet moves, requiring 14 time steps for its movement. Since the routing time in Existing Method 1 is the sum of the washing droplet movement time steps and the functional droplet movement time steps, it requires 20 time steps in total. Here, we refer to Existing Method 1 as “Existing Method (Waiting)” because the functional droplet waits for the washing droplet to finish moving.

**Figure 6:**
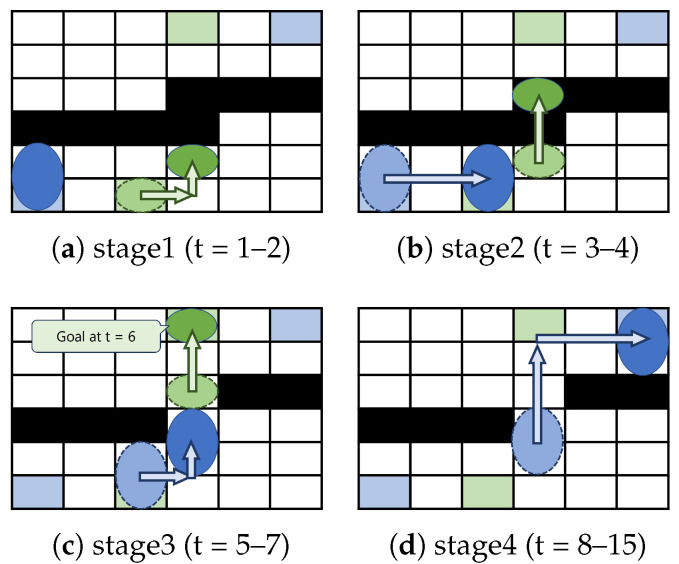
Example of Existing Method 2: (**a**) droplet routing step from time step 1 to time step 2; (**b**) droplet routing step from time step 3 to time step 4; (**c**) droplet routing step from time step 5 to time step 7; (**d**) droplet routing step from time step 8 to time step 15.

Next, we calculate the routing time based on Existing Method 2. In Existing Method 2, the washing droplet and the functional droplet move simultaneously. However, their movement paths are individually optimized. Since interference between the washing and functional droplets must be avoided, either droplet may need to pause when necessary. In Figure 6a, the movement of the functional droplet would interfere with the washing droplet, so only the washing droplet moves during time steps 1–2, after which the functional droplet can move. In Figure 6b, both the washing and functional droplets move simultaneously during time steps 3–4. At this point, the contaminated cells passed by the washing droplet are washed, allowing the functional droplet to pass. In Figure 6c, the washing droplet moves during time steps 5–6, while the functional droplet moves during time steps 5–7. In Figure 6d, the functional droplet moves during time steps 8–15. From the start to the end of droplet movement, Existing Method 2 requires 15 time steps in total. Here, we refer to Existing Method 2 as “Existing Method (Ignoring Velocity Differences)” because it determines routes without using a shape-dependent velocity model. In Existing Method (Ignoring Velocity Differences), each droplet is routed assuming that it takes 1 time step to move. However, in reality, movement takes more time due to the shape dependence of velocity.

Finally, we calculate the routing time based on the proposed method presented in this paper. In the proposed method, the washing droplet and the functional droplet are moved simultaneously. However, their paths are optimized by taking into account both the velocity differences caused by droplet shape and potential interference between the two droplets.

**Figure 7:**
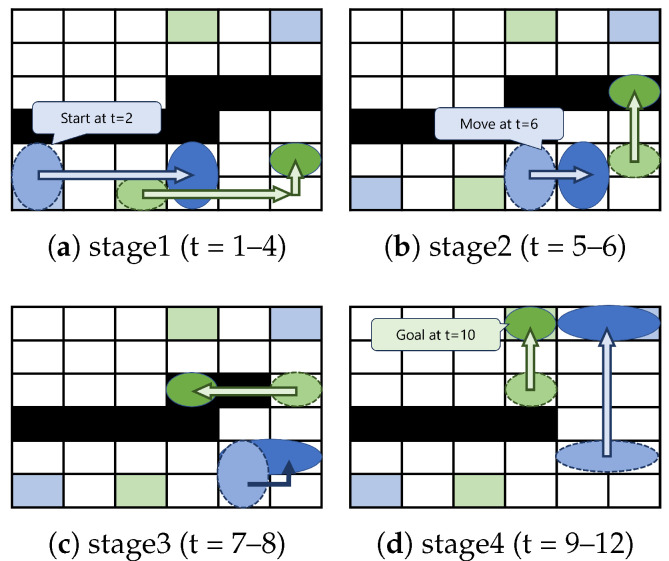
Example of the proposed method: (**a**) droplet routing step from time step 1 to time step 4; (**b**) droplet routing step from time step 5 to time step 6; (**c**) droplet routing step from time step 7 to time step 8; (**d**) droplet routing step from time step 9 to time step 12.

In Figure 7a, the washing droplet moves during time steps 1–4, while the functional droplet moves during time steps 2–4 to avoid interference. In Figure 7b, the washing droplet moves during time steps 5–6, and the functional droplet moves during time step 6 to avoid interference. In Figure 7c, the washing droplet moves during time steps 7–8, while the functional droplet morphs during time steps 7–8. At this point, the contaminated cells passed by the washing droplet are washed, allowing the functional droplet to pass through. In Figure 7d, the washing droplet moves during time steps 9–10, and the functional droplet moves during time steps 9–12.

The proposed method routes the droplets from their start cells to their goal cells in 12 time steps, a three-step improvement compared to Existing Method 2. Here, since the proposed method searches through all possible combinations, we define it as the Proposed Method (Exhaustive Search). Additionally, we propose an alternative version called the Proposed Method (Partial Constraints), which reduces the search space. This method is based on the idea proposed in [24], which states that the velocity-dependent optimization problem can be solved effectively by either

1. Moving along the chip edges in the direction where movement takes less time with only one shape-morphing operation;
2. Repeating diagonal movements and morphing operations only.

### 3.3. Formulation

We formulate the simultaneous routing with washing problem as an integer programming problem. Due to space limitations, the formulation is not fully linearized; however, equivalent constraints are described using logical expressions. Table 1 provides and defines the notation used in this formulation. In this formulation, we distinguish between steps and time. Steps refer to the number of operations performed, while time refers to the duration of those operations, measured in time steps. For example, when a droplet of size (w×h)=(1×2) moves one cell in the *x* direction, we describe it as requiring one step and taking two time steps to complete the movement.

We describe the coordinates and shapes of droplets using mathematical expressions. Let *i* denote the droplet index, which depends on the total number of droplets, and let *s* represent the operation step. The coordinates $\left({x.wash}_{i,s},{y.wash}_{i,s}\right)$ are defined as the reference point of washing droplet *i* at step *s*. Similarly, $\left(x_{i,s},y_{i,s}\right)$ is defined as the reference point of functional droplet *i* at step *s*. In this context, the reference point is the bottom-left corner of the droplet. The shape of functional droplet *i* at step *s* is expressed using $\left(w_{i,s},h_{i,s}\right)$, where $w_{i,s}$ and $h_{i,s}$ represent the width and height of the droplet, respectively. To reduce the electrical load on the electrodes, washing droplets are assumed to always be of size 1. Therefore, a washing droplet exists only at its reference point. In this paper, we assume that droplets always occupy rectangular regions of cells. Thus, functional droplet *i* occupies a cell area from $\left(x_{i,s},y_{i,s}\right)$ to $\left(x_{i,s}+w_{i,s}-1,\;y_{i,s}+h_{i,s}-1\right)$.

Formula (1) expresses that the washing droplet maintains a constant volume while morphing its shape. For example, if the droplet has a volume of 2, its shape at step *s* must be one of the following: $\left(w_{i,s}\times h_{i,s}\right)=\left(1\times 2\right)\;\mathrm{or}\;\left(2\times 1\right).$

(1) $$\begin{array}{c} \forall \;i,\;s,\;\;w_{i,s}\;\times \;h_{i,s}\;=\;{Vol}_{i} \end{array}$$

Given that the formulations for the washing droplet and functional droplet are the same from Formula (2) to Formula (5), we use the notation of ${dir.both}_{i,s}$ to refer to both ${dir.wash}_{i,s}$ and ${dir}_{i,s}$. Similarly, ${x.both}_{i,s}$ and ${y.both}_{i,s}$ represent the corresponding coordinates for the washing and functional droplets.

Formula (2) specifies the initial positions of the washing and functional droplets. The coordinates at step 0 for both types of droplet are provided as input.

(2) $$\begin{array}{c} \forall \;i,\;\;\left({x.both}_{i,0}\;=\;{X.Both.start}_{i}\right)\\ \wedge \;({y.both}_{i,0}\;=\;{Y.Both.start}_{i}) \end{array}$$

Next, we formulate the operations applied to droplets. In this study, we assume that the droplets may move in various directions or morph during routing. Droplets are allowed to move in the horizontal (*x*-axis), vertical (*y*-axis), and diagonal directions. The meaning of ${dir.both}_{i,s}$ for droplet *i* at step *s* is as follows:

1. When ${dir.both}_{i,s}=0$, the washing or functional droplet does not move in step *s*.
2. When ${dir.both}_{i,s}=1$, the droplet moves one cell in the horizontal direction.
3. When ${dir.both}_{i,s}=2$, the droplet moves one cell in the vertical direction.

In addition, for the functional droplet, ${dir}_{i,s}$ also includes the following:

1. When ${dir}_{i,s}=3$, the functional droplet morphs at step *s*.
2. When ${dir}_{i,s}=4$, the functional droplet moves one cell in the diagonal direction at step *s*.

Formula (3) represents the case when ${dir.both}_{i,s}=0$, indicating that neither washing droplet *i* nor functional droplet *i* moves at step *s*. This operation is primarily used to avoid interference between droplets.

(3) $$\begin{array}{cc} \forall \;i,\;s, & ({dir.both}_{i,s}\;=\;0)\;\to \\ & \left({x.both}_{i,s}\;=\;{x.both}_{i,s-1}\right)\;\wedge \;\left({y.both}_{i,s}\;=\;{y.both}_{i,s-1}\right) \end{array}$$

Formula (4) represents horizontal movement when ${dir.both}_{i,s}=1$. In this case, the reference points of washing droplet *i* and functional droplet *i* move in the horizontal direction, while the vertical coordinates remain unchanged.

(4) $$\begin{array}{cc} \forall \;i,\;s, & ({dir.both}_{i,s}\;=\;1)\;\to \\ & \left({x.both}_{i,s-1}-1\leq {x.both}_{i,s}\leq {x.both}_{i,s-1}+1\right)\\ & \wedge \;({y.both}_{i,s}\;=\;{y.both}_{i,s-1}) \end{array}$$

Similar to Formula (4), Formula (5) shows the vertical motion of ${dir.both}_{i,s}\;=\;2$:

(5) $$\begin{array}{cc} \forall \;i,\;s, & ({dir.both}_{i,s}\;=\;2)\;\to \\ & \left({x.both}_{i,s}\;=\;{x.both}_{i,s-1}\right)\\ & \wedge ({y.both}_{i,s-1}-1\leq {y.both}_{i,s}\leq {y.both}_{i,s-1}+1) \end{array}$$

Formula (6) describes the shape change of the droplet when ${dir}_{i,s}=3$. When droplet *i* morphs, its shape at step *s* is different from its shape at step s−1:

(6) $$\begin{array}{cc} \forall \;i,\;s, & ({dir}_{i,s}\;=\;3)\;\to \;\\ & \left(w_{i,s}\;\neq \;w_{i,s-1}\right)\;\wedge \;\left(h_{i,s}\;\wedge \;h_{i,s-1}\right) \end{array}$$

Formula (7) also shows the case of ${dir.both}_{i,s}\;=\;3$. Droplets can be reshaped in several ways, and the reference point changes depending on the type of reshaping. The expression that enables this possible reshaping is given by

(7) $$\begin{array}{ll} \forall \;i,\;s, & ({dir}_{s}\;=\;3)\;\to \end{array}\;\;\;\;\;\;\;\;\;\;\;\;\;\;\left\{\begin{array}{r} \left(x_{i,s}\leq x_{i,s-1}\right)\;\wedge \;\left(y_{i,s}\geq y_{i,s-1}\right)\\ \wedge (x_{i,s}+w_{i,s}\geq x_{i,s-1}+w_{i,s-1})\\ \wedge (y_{i,s}+x_{i,s}\leq y_{i,s-1}+h_{i,s-1}) \end{array}\right\}\;\;\;\vee \left\{\begin{array}{r} \left(x_{i,s}\geq x_{i,s-1}\right)\;\wedge \;\left(y_{i,s}\leq y_{i,s-1}\right)\\ \wedge (x_{i,s}+w_{i,s}\leq x_{i,s-1}+w_{i,s-1})\\ \wedge (y_{i,s}+x_{i,s}\geq y_{i,s-1}+h_{i,s-1}) \end{array}\right\}$$

Formula (8) represents diagonal movement when ${dir.both}_{i,s}=4$.

(8) $$\begin{array}{c} \forall \;i,\;s,\;({dir}_{i,s}\;=\;4)\;\to \;\\ \left(x_{i,s}\;=\;x_{i,s-1}ŷ1\right)\;\wedge \;\left(y_{i,s}\;=\;y_{i,s-1}ŷ1\right) \end{array}$$

Formula (9) shows whether the droplet has finished routing. Formula (9) determines if at least one cell of the droplet reaches the destination cell.


(9) $$\forall \;i,\;\sum_{s=1}\;\left\{\begin{array}{r} \left({x.both}_{i,s}\leq {X.goal}_{i}\right)\\ \wedge ({x.both}_{i,s}+w_{i,s}-1\geq {X.goal}_{i})\\ \wedge ({y.both}_{i,s}\leq {Y.goal}_{i})\\ \wedge ({y.both}_{i,s}+h_{i,s}-1\geq {Y.goal}_{i}) \end{array}\right\}$$

Formula (10) defines the list (${TList}_{i,s,d}$) that specifies the time required for each type of operation for functional droplet *i* at step *s* based on its current shape.

1. ${TList}_{i,s,0}$ represents the time required when no operation is performed (always one time step).
2. ${TList}_{i,s,1}$ represents the time required when the droplet moves in the *x*-axis (horizontal) direction.
3. ${TList}_{i,s,2}$ represents the time required when the droplet moves in the *y*-axis (vertical) direction.
4. ${TList}_{i,s,3}$ represents the time required when the droplet moves diagonally.
5. ${TList}_{i,s,4}$ represents the time required when the droplet undergoes shape deformation.

(10) $$\begin{array}{cc} \forall \;i,\;s, & \left({TList}_{i,s,0}\;=\;1\right)\\ & \wedge ({TList}_{i,s,1}\;=\;w_{i,s})\\ & \wedge ({TList}_{i,s,2}\;=\;h_{i,s})\\ & \wedge ({TList}_{i,s,3}\;=\;Max(w_{i,s},h_{i,s}))\\ & \wedge ({TList}_{i,s,4}\;=\;|w_{i,s}-h_{i,s}+1|) \end{array}$$

Formula (11) calculates the routing time (${step2time}_{i,s}$) for functional droplet *i* at operation step *s*. It adds the time required for the current operation, determined by the direction (${dir}_{i,s}$), to the routing time of the previous step (${step2time}_{i,s-1}$).

(11) $$\begin{array}{c} \forall \;i,\;s,\;\;{step2time}_{i,s}\;=\;{step2time}_{i,s-1}\;+\;{TList}_{i,s,{dir}_{i,s}} \end{array}$$

Formula (12) calculates the routing time (${step2time.wash}_{i,s}$) for washing droplet *i* at operation step *s*. Since the volume of a washing droplet is always 1 in this problem, the routing time and the operation step are always equal.

(12) $$\begin{array}{c} \forall \;i,\;s,\;\;{step2time.wash}_{i,s}\;=\;s \end{array}$$

The constraint related to unavailable cells is provided in Formula (13). A functional droplet is not allowed to enter an unavailable cell. However, it may enter the cell after a washing droplet has passed through and washed it.

(13) $$\begin{array}{c} \forall \;i,\;s,\;t,\; \end{array}\;\;\;\neg \left\{\begin{array}{r} \left(x_{i,s}\leq Contami.x\right)\\ \wedge (x_{i,s}+w_{i,s}-1\geq Contami.x)\\ \wedge (y_{i,s}\leq Contami.y)\\ \wedge (y_{i,s}+h_{i,s}-1\geq Contami.y) \end{array}\right\}\;\;\;\vee \;\sum_{s=1}\left\{\begin{array}{r} \left(s<{step2time}_{i,s}\right)\\ \wedge ({x.wash}_{i,s}=Contami.x)\\ \wedge ({y.wash}_{i,s}=Contami.y) \end{array}\right\}$$

Formulae (14) and (15) are the timing constraints. Since there is no difference in the formulae between washing and functional droplets, the ${time.both}_{i}$ variable represents both ${time.wash}_{i}$ and ${time}_{i}$, and ${step2time.both}_{i,s}$ represents both ${step2time.wash}_{i,s}$ and ${step2time}_{i,s}$.

Formula (14) defines the routing time (${time.both}_{i}$) for droplet *i*.


(14) $$\forall \;i,\;\;{time.both}_{i}\;=\;Max\{({step2time.both}_{i,s})\}l$$

The objective function minimizes the maximum routing time among all functional and washing droplets.

(15) $$Minimize:{Max}_{i}\;\left({time.both}_{i}\right)$$

Additionally, in the Proposed Method (Partial Constraints), Formula (16) is added to the above formulation.

(16) $$\begin{array}{cc} \forall \;i,\;s, & ({dir}_{i,s}\;=\;1)\;\wedge \;\left(x_{i,s}+w_{i,s}-1\neq W\right)\\ & \vee ({dir}_{i,s}\;=\;2)\;\wedge \;(y_{i,s}+h_{i,s}-1\neq H)\;\to \\ & \left({dir}_{i,s+1}\;=\;{dir}_{i,s}\right) \end{array}$$

## 4. Experiments

### 4.1. Setup

We experimentally demonstrated the effectiveness of the proposed method for the simultaneous routing of washing and functional droplets, comparing existing methods with the proposed method. The input parameters included the biochip size, the positions of contaminated cells, the volumes of the washing and functional droplets, and their respective start and goal cells. The objective function minimizes the maximum routing time of droplets from their start cells to their goal cells. The following four methods were compared:

- Existing Method (Wait): The functional droplet starts moving only after the washing droplet is done moving [23].
- Existing Method (Ignoring Velocity Differences): The washing and functional droplets move simultaneously, without using a shape-dependent velocity model.
- Proposed Method (Exhaustive Search): The washing and functional droplets move simultaneously, with a shape-dependent velocity model used.
- Proposed Method (Partial Constraints): A variation of the exhaustive search method that restricts the functional droplet’s movement directions to reduce the solution space.

The Existing Method (Wait) uses the formulation in Section 3.3, and the functional droplet cannot move until ${Max}_{i}\left({time.wash}_{i}\right)$.

The Existing Method (Ignoring Velocity Differences) uses the formulation in Section 3.3, but Formula (10) is exchanged for Formula (17).


(17) $$\begin{array}{cccc} \forall \;i,\;s, & \left({TList}_{i,s,0}\;=\;1\right)\\ & \wedge ({TList}_{i,s,1}\;=\;1) & & \wedge ({TList}_{i,s,2}\;=\;1)\\ & \wedge ({TList}_{i,s,3}\;=\;1) & & \wedge ({TList}_{i,s,4}\;=\;1) \end{array}$$

The experimental conditions were as follows:

- The washing droplet size was set to 1, with only one such droplet present on the biochip.
- The functional droplet size was set to 2, with only one present on the biochip.
- The biochip size is assumed to be W=10,H=10.
- Experiments were conducted for two contamination ratios: 10% and 20% of the total biochip cells.
- Contaminated cells were assigned randomly in each scenario.
- The start and goal cells of the washing droplet were randomly assigned for each scenario with certain constraints: the start cell’s *y*-coordinate was 1, and its goal cell’s *y*-coordinate was *H*.

For each contamination ratio, 30 different scenarios were tested by varying the start and goal cells of the washing droplet, and only cases for which a solution was found are shown in the graphs.

The experiments used a Ryzen Threadripper 3970X (3.7 GHz, 32 cores, 64 threads) and 256 GB of memory. IBM ILOG CPLEX Optimization Studio 20.1.0 was used to find a solution with each of the four methods: the existing methods and the proposed methods. The computation time was limited to a maximum of 10 h of CPU time. If an optimal solution was not obtained within the time limit, the best feasible solution found within that time was used for comparison.

### 4.2. Results

**Figure 8:**
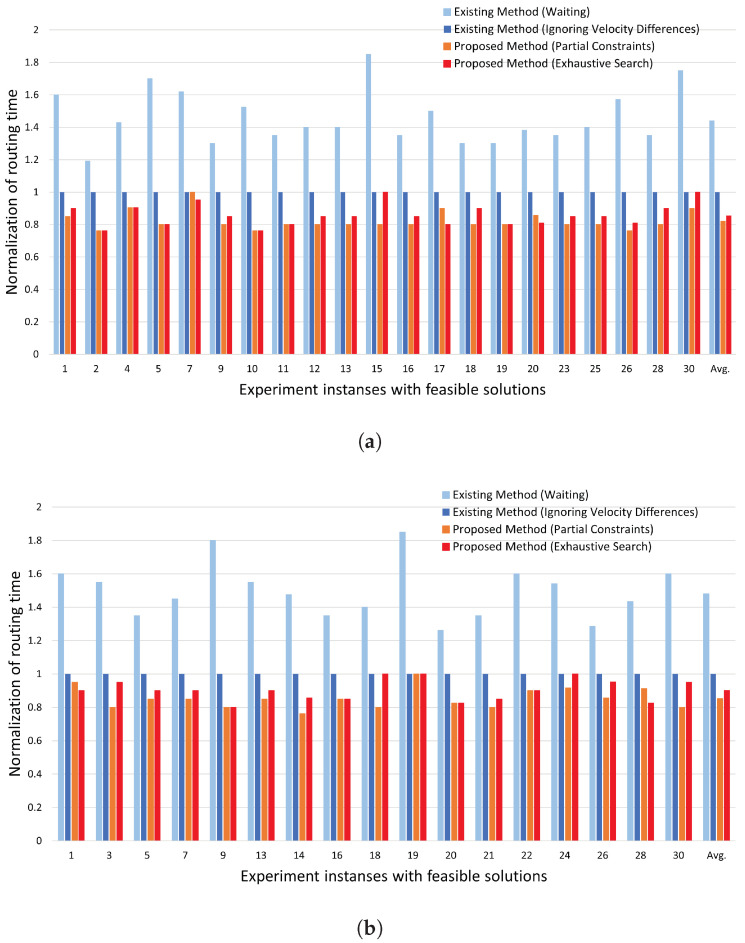
Results: (**a**) 10% contaminated cell ratio; (**b**) 20% contaminated cell ratio. (**a**) Results for 10%. The proposed method (exhaustive search) achieved an average reduction in routing time of 10%. The proposed method (partial constraints) achieved an average reduction in routing time of 20%. (**b**) Results for 20%. The proposed method (exhaustive search) achieved an average reduction in routing time of 10%. The proposed method (partial constraints) achieved an average reduction in routing time of 15%.

Figure 8a,b show the experimental results for contaminated cell ratios of 10% and 20%, respectively. The horizontal axis represents the problem indices for which solutions are obtained, and the vertical axis shows the routing time achieved with each method, normalized by the routing time of the existing method (ignoring velocity differences). The rightmost bar in each graph represents the average of all the obtained results.

In all test cases, the two proposed methods achieved shorter routing times compared to both existing methods. As shown in Figure 8, the proposed methods successfully reduced the routing time by using a shape-dependent velocity model, allowing the functional droplet to move simultaneously with the washing droplet. In comparison to the Existing Method (Ignoring Velocity Differences),

1. The proposed method (exhaustive search) achieved an average reduction in routing time of 10% for contamination ratios of 10% (Figure 8a) and 20% (Figure 8b).
2. The proposed method (partial constraints) achieved an average reduction in routing time of 20% for the 10% contamination ratio (Figure 8a and a reduction in routing time of 15% for the 20% contamination ratio (Figure 8b).

We now discuss the difference between the full-search and partial-constraint results. The improved performance of the partial constraint method is attributed to the reduced solution space, which was achieved by imposing additional movement restrictions on the functional droplet. This reduction in complexity allowed for more efficient exploration and solution optimization.

Like contaminated cells, faulty cells can be considered unavailable cells [25]. However, the proposed method assumes that unavailable cells can be resolved with the passage of a washing droplet. Therefore, the method cannot be applied to cases involving faulty cells. The resolution of problems involving both faulty and contaminated cells remains a subject for future work.

## 5. Conclusions

In this paper, we propose a simultaneous routing method for washing and functional droplets on a MEDA biochip, aiming to minimize the routing time of functional droplets in MEDA biochips using a shape-dependent velocity model. Compared to existing methods, the proposed approach successfully reduced the droplet routing time by an average of 10%. In future work, we plan to extend the proposed method to address challenges such as washing capacity limits and faulty cells.

## Figures and Tables

**Table 1:** Notation.

Character	Meaning	Ranges
Vol	Volume of functional droplet	Given
Wash.Num	Number of washing droplets	Given
Num	Number of functional droplets	Given
$\left(\;{X.Wash.start}_{i}\;,\;{Y.Wash.start}_{i}\;\right)$	Start cell of washing droplet *i*	Given, 1≤i≤Wash.Num
$\left(\;{X.Wash.goal}_{i}\;,\;{Y.Wash.goal}_{i}\;\right)$	Goal cell of washing droplet *i*	Given, 1≤i≤Wash.Num
$\left(\;{X.start}_{i}\;,\;{Y.start}_{i}\;\right)$	Start cell of functional droplet *i*	Given, 1≤i≤Num
$\left(\;{X.goal}_{i}\;,\;{Y.goal}_{i}\;\right)$	Goal cell of functional droplet *i*	Given, 1≤i≤Num
(Contami.x,Contami.y)	Coordinates of contaminated cell	Given
$\left(\;{x.wash}_{i,s}\;,\;{y.wash}_{i,s}\;\right)$	Reference point of washing droplet *i* at step *s*	$1\;\leq \;{x.wash}_{i,s}\;\leq \;W\;,\;1\;\leq \;{y.wash}_{i,s}\;\leq \;H$
$\left(\;x_{i,s}\;,\;y_{i,s}\;\right)$	Reference point of functional droplet *i* at step *s*	$1\;\leq \;x_{i,s}\;\leq \;W\;,\;1\;\leq \;y_{i,s}\;\leq \;H$
$\left(\;w_{i,s}\;,\;h_{i,s}\;\right)$	Width and height of functional droplet *i* at step *s*	$1\;\leq \;(\;w_{i,s}\;,\;h_{i,s}\;)\;\leq \;Vol$
${dir.wash}_{i,s}$	Operation of washing droplet *i* at step *s*	$0\;\leq \;{dir.wash}_{i,s}\;\leq \;2$
${dir}_{i,s}$	Operation of functional droplet *i* at step *s*	$0\;\leq \;{dir}_{i,s}\;\leq \;4$
${step2time.wash}_{i,s}$	Elapsed time of washing droplet *i* until step *s*	$0\;\leq \;{step2time.wash}_{i,s}$
${step2time}_{i,s}$	Elapsed time of functional droplet *i* until step *s*	$0\;\leq \;{step2time.}_{i,s}$
${time.wash}_{i}$	Routing time of washing droplet *i*	$0\leq {time.wash}_{i}$
${time}_{i}$	Routing time of functional droplet *i*	$0\leq {time}_{i}$

## Data Availability

The original contributions presented in this study are included in this article. Further inquiries can be directed to the corresponding author.
